# Assessing the Potential of Fecal NIRS for External Marker and Digestibility Predictions in Broilers

**DOI:** 10.3390/ani15152181

**Published:** 2025-07-24

**Authors:** Oussama Tej, Elena Albanell, Ibtissam Kaikat, Carmen L. Manuelian

**Affiliations:** 1 Group of Ruminant Research (G2R), Department of Animal and Food Science, Autonomous University of Barcelona (UAB), 08193 Bellaterra, Spain; oussama.tej@irta.cat (O.T.); carmen.manuelian@uab.cat (C.L.M.); 2 Animal Nutrition Program, Institute of Agrifood Research and Technology (IRTA), 43120 Constantí, Spain; 3 Animal Nutrition and Welfare Service (SNiBA), Department of Animal and Food Science, Autonomous University of Barcelona (UAB), 08193 Bellaterra, Spain; ibtissam.kaikat@uab.cat

**Keywords:** NIRS, broiler, digestibility marker, PEG, excreta

## Abstract

This study evaluated fecal near-infrared spectroscopy (fNIRS) potential to predict three external markers (Yb, Ti, and polyethylene glycol (PEG)) and dry matter digestibility (DMD) calculated from these markers and fiber fractions. A total of 192 fecal samples were collected from 576 Ross 308 male chicks supplemented with TiO_2_ (2 g/kg), Yb_2_O_3_ (50 mg/kg), and PEG (5 g/kg) for 8 d. Reference values for Ti and Yb were obtained using an inductively coupled plasma–optical emission spectrometer, for fiber fractions via ANKOM, and for PEG content using an ad hoc fNIRS model. Prediction models were developed in external validation with 25% of the samples. Good and fair prediction models were built for Ti and Yb, respectively, and considered adequate for rough screening. The DMD models based on Yb and ADF were unreliable, whereas the model based on Ti was suitable for rough screening. The PEG prediction model built during the adaptation period performed exceptionally well; however, the DMD prediction based on PEG highlighted limitations due to diet differences during both the adaptation and experimental periods. In conclusion, fNIRS shows promise for screening Ti and Yb fecal content and DMD using Ti. However, tailored PEG prediction equations need to be developed for each specific diet.

## Simple Summary

This study investigated whether fecal near-infrared spectroscopy (fNIRS) could be used to estimate feed digestibility in broiler chickens, an application that remains underexplored. Chicks were fed diets containing external markers, ytterbium (Yb), titanium (Ti), and polyethylene glycol (PEG) to assess dry matter digestibility (DMD) and fiber-based DMD. The results showed that fNIRS could predict Ti and Yb concentrations in excreta and DMD based on Ti, suggesting its potential as a rapid screening tool for digestibility. However, DMD predictions based on PEG were less reliable due to variations in diet. These findings suggest that fNIRS could enhance feed evaluation in broilers; however, specific PEG calibration models are required for different diets.

## 1. Introduction

Optimizing the digestive efficiency of poultry is a promising strategy to minimize nutrient losses and improve feed utilization [1]. Among the various methods used to assess digestibility in poultry, the in vivo method based on indigestible markers (internal and external) plays an important role in accurately evaluating nutrient utilization in avian systems. These markers offer insight into the passage and utilization of nutrients in the poultry digestive tract, facilitating easier and more accurate digestibility evaluation.

External markers such as chromium oxide (Cr_2_O_3_) and titanium dioxide (TiO_2_) have been widely used to assess the metabolizability and digestibility of diets and feed ingredients in broilers [2,3]. Internal markers, including indigestible fibers, acid-insoluble ash, and n-alkanes, have been evaluated in broilers, laying hens, and turkeys [4,5]. However, determining these markers using wet chemistry presents several challenges as it is labor-intensive, time-consuming, and requires highly skilled personnel, along with the use of genotoxic reagents. For instance, Cr_2_O_3_ is no longer used in digestibility studies due to its toxic and carcinogenic effects on laboratory workers during analytical procedures [6], and the European Food Safety Authority has recently banned TiO_2_ due to genotoxicity risks [7]. Alternatively, rare earth elements such as ytterbium (Yb) in the form of ytterbium oxide (Yb_2_O_3_) have been studied in cattle and pigs with excellent results [8,9]. Moreover, polyethylene glycol (PEG) has also been suggested as a safe external marker for ruminants because it is not degraded during digestion, and the recovery rate exceeds 95% [6,10]. Additionally, PEG content can be determined with ad hoc prediction models based on near-infrared reflectance spectroscopy (NIRS) [11]. This technique could be also explored to predict various other organic and inorganic markers, further expanding its application in digestibility studies as a direct method to estimate nutrient digestibility directly from feces [12,13] and as an indirect method by estimating indigestible markers content from feces, which are often used in digestibility studies to replace methods based on total fecal sampling [14]. This application is known as fecal NIRS (fNIRS).

The feasibility of fNIRS has been studied in domestic and wild ruminants using internal and external markers but remains limited in poultry and monogastrics in general [15]. Studies have shown that fNIRS effectively quantifies internal markers, such as alkanes and indigestible fiber, and estimates dry matter intake (DMI) and digestibility (DMD) in various livestock species, including dairy and beef cattle [16,17], sheep [18], goats [19], and horses [20]. Synthetic n-alkanes were among the first external markers tested using fNIRS, but they require high doses and purified forms [21]. In rats and poultry, the application of fNIRS to predict Cr_2_O_3_ content has also been studied for various feeds [22]. Moreover, PEG determined with NIRS has shown promise for estimating fecal excretion in goats [11], cattle [14,23], and dairy sheep [24], as well as for assessing dry forage and rangeland intake [10,25] and organic matter intake [6] in small ruminants. In these studies, PEG was administered orally by dosing with a drenching gun or similar. To the best of our knowledge, only one study has evaluated fNIRS for predicting DMD, starch, and protein digestibility in poultry, achieving low model performance [26].

Based on previous studies in ruminants and pigs, we hypothesized that fNIRS could serve as a valuable tool for predicting DMD from external (Yb, Ti, and PEG) and internal (fiber fractions) markers in broilers. Therefore, this study aimed to investigate the feasibility of fNIRS to determine Yb and Ti content in broiler excreta samples and, also, to assess the potential of fNIRS to predict DMD based on Yb, Ti, PEG, and fiber fractions in poultry.

## 2. Materials and Methods

### 2.1. Samples Origin

A total of 192 fecal samples from 576 male chicks of the Ross 308 strain, allotted in 96 battery brooder cages with 6 birds per cage, were obtained from a previous study [27] conducted by the Animal Nutrition and Welfare Service (SNiBA) of the Autonomous University of Barcelona (UAB; Bellaterra, Spain). The study lasted 25 d, taking place between May and June 2022. Briefly, chicks were fed 32 different experimental diets (Table 1) formulated with 3 cereals (wheat, barley, and rye), and for each cereal 4 different genotypes, with and without enzyme supplementation (a mix of β-glucanase, phytase, and xylanase at doses of 20,000 BU/kg, 1000 FTU/kg, and 16,000 BXU/kg, respectively), 6 processed proteins, and 2 feather meal feeds. All of these provided the variability needed to develop NIRS predictive models. In addition, each diet was supplemented with TiO_2_ at 2 g/kg, Yb_2_O_3_ at 50 mg/kg, and PEG at 5 g/kg as external markers for later determination of digestibility. The markers were finely ground to a particle size of 1 mm to ensure their uniform distribution throughout the diets and to prevent diet selection.

From day 1 to 15, all chicks received an adaptation diet; then, from day 16 to 25, they were fed the corresponding experimental diet (Table 1). Excreta were collected over a continuous 24 h period on days 20 and 25 of the experiment, with six replicate samples collected per treatment. Clean trays were placed under each cage 24 h before sampling. During collection, meticulous care was taken to exclude any impurities, such as feed or feathers. These samples were subsequently dried at 60 °C for 48 h until a constant weight was achieved, following the AOAC #2001.12 method [28]. After drying, fecal samples were ground, sifted through a 1 mm sieve, and then stored in plastic bags at 4 °C. These samples were set aside until chemical and spectral analysis were conducted at the laboratory unit of the Department of Animal and Food Sciences at the UAB.

### 2.2. Reference Analysis

Ytterbium and Titanium were quantified following the AOAC #984.27 method [28] at the Servei d’Anàlisi Química at the UAB using an inductively coupled plasma–optical emission spectrometer (5900 ICP-OES; Agilent, Santa Clara, CA, USA). Before the ICP-OES determination, dried and ground fecal samples underwent a digestion using concentrated nitric acid (HNO_3_) and tetrafluoroboric acid (HBF_4_), diluted with HNO_3_ 1% (*v*/*v*) before being injected.

The chemical analyses of excreta were carried out in duplicate. Dry matter was determined by heating at 103 °C for 24 h. Fiber fractions were analyzed for the subset of fecal samples collected from cereal-fed chicks (*n* = 144). Sequential analysis for neutral detergent fiber (NDF), acid detergent fiber (ADF), and acid detergent lignin (ADL) was performed using the ANKOM 200/220 fiber analyzer (Ankom tech. Co., Fairport, NY, USA) according to the ANKOM methodology. The analysis was performed on an ash-free basis using α-amylase and without the addition of sodium sulfite (Na_2_SO_3_). Lignin content was determined by dissolving cellulose with sulfuric acid according to the AOAC #973.18 method [28].

To quantify PEG in excreta, since a direct laboratory method is unavailable, NIRS ad hoc prediction models were developed by adding known increasing amounts of PEG to fecal samples, following the indications of Landau et al. [11]. The fecal samples used to develop the calibration model were collected during the adaptation period, i.e., before the introduction of the experimental diets. Fecal samples were dried in a forced-draft oven at 60 °C for 48 h, ground through a 1 mm sieve, and pooled. Twenty-seven fortified samples with PEG concentrations ranging from 0% to 15% were obtained by adding 1% increments of PEG. To ensure PEG homogeneity with the sample, 20 mL of bi-distilled water was added to the mixtures and was allowed to react for 3 d at room temperature (24 °C). Afterward, the mixtures were dried, ground, and kept in a dry state until the spectra were collected. The NIRS procedure for scanning the samples and developing the PEG prediction model is explained in the following subsections. The PEG prediction model was then applied to the 192 spectra collected from the excreta samples to determine their PEG content.

The DMD was then determined for each marker according to the following formula:

$$\mathrm{DMD}\;=\;1-\frac{\%\;Marker\;in\;diet}{\%\;Marker\;in\;excreta}$$

### 2.3. Spectral Collection

Approximately 2 g of each dried and ground sample was scanned on a spectrophotometer equipped with a scanning monochromator with a spectral range of 1100 to 2500 nm (NIRS 5000; FOSS NIRSystems, Hillerød, Denmark). Spectral data were collected at 2 nm intervals, resulting in 692 data points. Measurements were performed in reflectance mode in a small circular quartz glass cup (48 mm diameter). Samples were scanned in duplicate (two different cup fillings). The collected spectra were averaged to achieve higher model accuracy, and absorbance was recorded as log(1/R), where R represents the reflected energy.

### 2.4. Chemometric Analysis

Chemometric models were developed for PEG, Yb, and Ti contents in excreta, and the DMD coefficients were calculated from Ti, Yb, PEG, and ADF using WinISI III v. 4.10 software (Infrasoft International; Port Matilda, PA, USA) with modified partial least-squares (MPLS) analysis. The calibration models optimized with a 6-fold cross-validation were built using 75% of the samples and validated externally with the remaining 25% of the samples. Two passes of chemical (t) and spectral (H) outliers were performed to eliminate samples with a critical T-value of ≥2.5, which removed samples with a high discrepancy between predicted and reference values, and a critical H-value of ≥10.00, which eliminated samples with markedly different spectra. Outliers never represented >10% of the total samples used.

To build the calibration models, different combinations of scatter corrections (SNV, standard normal variate; D, detrend; SNV + D; MSC, multiplicative scatter correction) to reduce the effects of particle size were combined with four mathematical treatments (1.4.4.1, 2.4.4.1, 1.5.5.1, and 2.5.5.1; where the first digit is the number of the derivative, the second is the gap over which the derivative is calculated, the third is the number of data points in a running average or smoothing, and the fourth is the second smoothing). The best prediction models were selected based on the highest coefficient of determination for calibration (R^2^_CAL_), cross-validation (R^2^_CV_), and external validation (R^2^_VAL_); the lowest standard error of calibration (SEC), cross-validation (SECV) and standard error of prediction (SEP); bias close to zero and slope close to one; ratio of error to range (RER) ≥ 10, with RER representing the ratio of the data range of the validation samples to the SEP value; and ratio of performance to deviation (RPD) ≥ 3, where RPD is defined as the ratio of the standard deviation of the validation samples to the SEP value [29,30]. If RPD did not reach the minimum threshold of 3, according to Saeys et al. [31], values between 2.0 and 2.5 can still be used to establish acceptable quantitative predictions for specific compounds.

## 3. Results

### 3.1. Ad Hoc PEG Calibration in Excreta

**Figure 1:**
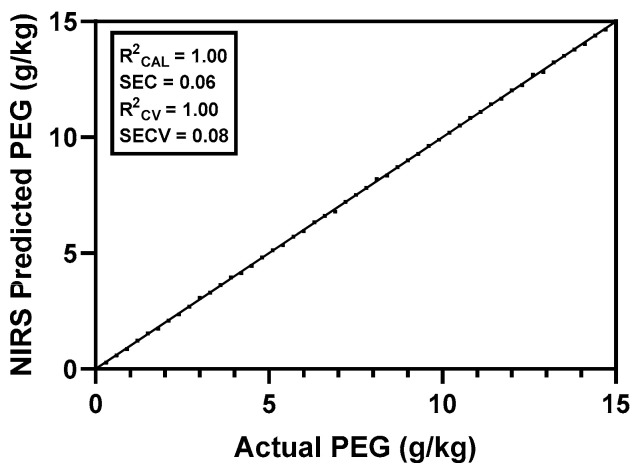
Performance of the near-infrared spectroscopy (NIRS) calibration model for polyethylene glycol (PEG) content in excreta samples using increasing concentrations ranging from 0 to 15% (*n* = 51). R^2^_CAL_, coefficient of determination of calibration; SEC, standard error of calibration; R^2^_CV_, coefficient of determination of cross-validation; SECV, standard error of cross-validation.

The best calibration model was achieved with SNV for scatter correction combined with the mathematical treatment 1.4.4.1. The performance of the developed model is depicted in Figure 1, demonstrating excellent results with a perfect R^2^_CAL_ (1.00) and R^2^_CV_ (1.00), indicating that the model explains all the variance in both the calibration and cross-validation sets. Additionally, the low values for SEC (0.06) and SECV (0.08) reflect the model’s precision and robustness, suggesting minimal prediction error.

### 3.2. NIRS Predictive Models to Quantify Yb and Ti

Overall, the fecal samples collected showed moderate variability for both Yb and Ti, with a coefficient of variation (CV) of approximately 11%. Notably, both markers exhibited similar characteristics in terms of range, mean, and standard deviation between the calibration and validation datasets, as shown in Table 2, which is crucial for developing accurate predictive models.

The optimal calibration models for Ti and Yb were attained by employing the second derivative in combination with SNV+D and MSC as scatter correction techniques (Table 3). The outliers removed were 8.16% for Ti and 4.77% for Yb, which is within the previously established threshold. The R^2^_CAL_ of the two models showed a strong relationship (>0.87) between the reference data and the NIRS-predicted values for Yb and Ti markers. However, R^2^_CV_ (>0.74) and R^2^_VAL_ (>0.67) were slightly lower than R^2^_CAL_ (Table 3). Furthermore, the bias values revealed no systematic deviation between the predicted and reference values for both markers, with slopes of 0.89 for Ti and 0.86 for Yb (Table 3). In both cases, RER values were ≥10, but a better RER was observed for Yb than for Ti, probably due to the lower SEP in the former than the latter. The RPDs for Ti and Yb were found to be below the threshold of 2.4, with RPD values of 2.3 and 2.0, respectively.

### 3.3. NIRS Predictive Models to Quantify Digestibility

**Figure 2:**
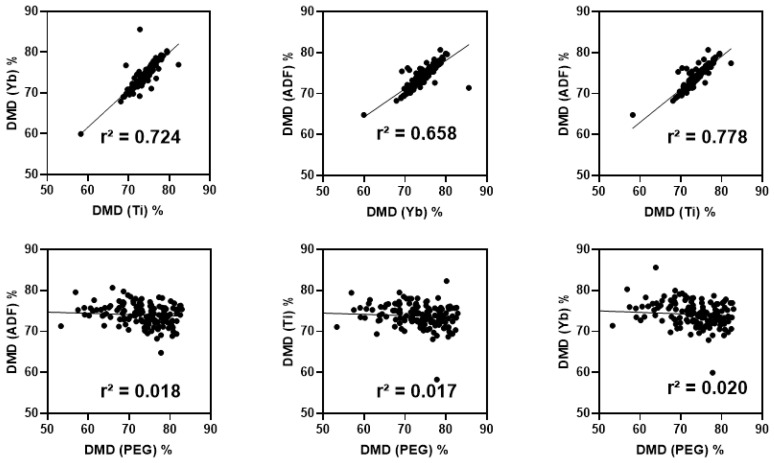
Linear regression plot of dry matter digestibility (DMD, %) based on Yb, Ti, polyethylene glycol (PEG), and acid detergent fiber (ADF) (*n* = 144).

Digestibility coefficients were calculated using external indigestible markers (Yb, Ti, and PEG predicted content) and only ADF as internal markers, since NDF and ADL showed incomplete recovery. Reference DMD results calculated using these markers revealed moderate correlations, indicating a good association only between DMD_Yb_, DMD_Ti_, and DMD_ADF_ (r^2^ ≥ 0.66; Figure 2), confirming the reliable use of these three markers for digestibility assessment. However, the correlation of DMD_Yb_, DMD_Ti_, and DMD_ADF_ with DMD_PEG_ was found to be null (r^2^ ≤ 0.02; Figure 2). Based on this low correlation, it was decided to develop prediction models for digestibility only, using Yb, Ti, and ADF as predictors.

The calibration and validation databases showed similar ranges for digestibility based on three markers: Yb, Ti, and ADF. Descriptive statistics for DMD calibration and validation databases are shown in Table 2, whereas the performance of prediction models is displayed in Table 3. The most effective predictive model for all DMD markers was achieved using the second derivative. To correct the scatter, the optimal approach was SNV + D for DMD_Yb_ and DMD_ADF_, and MSC for DMD_Ti_. The outliers removed were 5.44%, 4.08%, and 7.82% for DMD_Ti_, DMD_Yb_, and DMD_ADF,_ respectively, all of which remain below the established threshold.

Based on R^2^_CAL_ values, precise calibrations for DMD were established for all three markers, as evidenced by R^2^_CAL_ values of 0.89, 0.91, and 0.90 for DMD_Yb_, DMD_Ti_, and DMD_ADF_, respectively (Table 3). In cross-validation, good predictive ability for DMD_Ti_ and DMD_Yb_ was obtained with R^2^_CV_ values of 0.80 and 0.75, respectively. However, the R^2^_CV_ obtained for DMD_ADF_ (R^2^_CV_ = 0.66) was relatively low (Table 3). Moreover, SECV values were slightly higher than the corresponding SEC values for all three parameters (Table 3). The difference was more pronounced for DMD_Yb_ and DMD_ADF_, exceeding 50%, while for DMD_Ti_, it was 34%. The model’s performance in external validation slightly dropped for DMDTi and DMDYb, with R^2^_VAL_ values of 0.77 and 0.68, respectively (Table 3). The bias values for DMD_Yb_ and DMD_Ti_ were consistently low, measuring 0.02 and 0.01, respectively. Moreover, the slope values for both markers were close to 1, with values of 0.86 and 0.90, respectively, indicating good accuracy. The RER values for DMD_Yb_ and DMD_Ti_ exceeded the minimum recommended threshold of 10, with values of 15.2 and 18.6, respectively. DMDTi exhibited the greatest RPD, reaching 2.4. In contrast, the prediction model for DMD_ADF_ performed poorly, with an R^2^_VAL_ of 0.25, an RPD of 1.2, and biases and slopes far from 0 and 1, respectively.

## 4. Discussion

### 4.1. Feasibility of PEG in Broilers for Digestibility

The developed NIRS equation for PEG in broiler excreta, achieving an outstanding R^2^_CV_ of 1 and an SECV of 0.08, suggests the applicability of PEG as an external marker in broilers, despite its lower concentration compared to ruminant studies. Previous studies in ruminant species demonstrated PEG’s effectiveness in goats [11,32], dairy ewes and sheep [6,10,24], and cows [14,22] as an external marker with R^2^_CV_ ≥ 0.988 and SECV ≤ 3.5 for estimating feed intake capacity or fecal production. In monogastric species, only one study evaluated PEG’s efficacy as an indigestible marker for assessing digestive ability in growing pigs [33], which also reported R^2^_CV_ and R^2^_VAL_ ≥ 0.99. None of these studies have reported any alterations in metabolism, intake, or digestibility attributable to PEG.

Although the calibration model for PEG concentration in broiler excreta demonstrated exceptional accuracy, precise quantification does not necessarily ensure a reliable assessment of digestibility. In agreement with a study on growing pigs [33], our predictive models failed to accurately predict PEG content and DMD, as shown by the low correlation between DMD_PEG_ and reference DMD calculated from Yb, Ti, and ADF. These authors also reported that the recovery of PEG varied significantly across different diets and periods, with the correlation between predicted and theoretical PEG concentrations being insufficient (<50%), especially when pigs were fed a low-fiber diet with higher marker concentrations. Additionally, PEG recovery rates were influenced by the breed of pigs. Although tannin levels are generally low in poultry feed because they produce adverse effects on feed intake, nutrient digestibility, and growth performance in chickens [34], the existing literature has documented that PEG may not function optimally in diets containing high levels of specific compounds such as polyphenols [11,35], which can alter the digestibility and intake capacity. Landau et al. [32] demonstrated that the presence of tannins in goats’ diets reduced the recovery rate of PEG from 97.8% to 42.7% after animals were given PEG at a dose of 14 g/day. All of these findings underscore that PEG’s efficacy as an external digestibility marker can be predicted with the NIRS method, but its accuracy is highly dependent on diet composition and calibration conditions. Therefore, we hypothesized that our PEG calibration failed to determine DMD due to the diet-dependent nature of PEG calibration, as the calibration equation for PEG was developed ad hoc using fecal samples collected before the animals started the experimental diet. 

### 4.2. Accuracy of NIRS Models for Yb, Ti, and Fiber Fractions, and DMD

The moderate CV (≈11%) observed for Yb and Ti concentrations reflects the biological and dietary diversity among the fecal samples used, which originated from different cereal types, genotypes, and enzyme treatments, which, in turn, helps develop more accurate prediction models [36]. For both markers, the calibration and validation datasets presented similar characteristics in terms of range, mean, SD, and CV (Table 2), which is crucial to developing accurate predictive models [36]

The best prediction models were achieved using the second derivative, which enhanced peak resolution for more precise modeling [37]. The SNV normalized spectra to eliminate additive and multiplicative scattering effects, while MSC used a reference spectrum for multiplicative correction. The SNV+D, with its detrending step, further improved scatter correction by removing baseline variations before normalization, making it more effective for complex samples [38].

The precision of all calibration models based on the R^2^_CV_ could be considered adequate for approximate quantitative prediction, as suggested by Karoui et al. [39], who reported R^2^_CV_ values ranging from 0.66 to 0.81. Despite R^2^_VAL_ being lower than R^2^_CV,_ they were still in the range for approximate quantitative prediction, except for DMD_ADF_, which is considered unsatisfactory. The lower R^2^_VAL_ values compared to R^2^_CAL_ and R^2^_CV_ are common when developing prediction models in external validation because they consider new samples not included during calibration. This may have introduced slight spectral discrepancies, which could affect prediction accuracy. The slopes for Yb, Ti, DMDYb, and DMDTi suggest potential limitations of the prediction models, as those with values ±0.15 (0.85–1.15) indicate lower accuracy at the extremes [40]. The RPD values found for Yb, Ti, and DMD_Ti_ suggest that these models can only be considered suitable for rough screening purposes [29]. Generally, the RER is larger than the RPD by a factor of approximately 4 or 5; however, there is no simple conversion factor between them, and their relationship depends on the sample distribution [41]. The RPD is preferred over RER to assess the quality of the prediction model because it considers the expected range and is less affected by extreme samples in the validation set [41].

The low accuracy of the developed models could be related to the low amount of Yb, Ti, and ADF in the diet, as the concentration of a component is a crucial factor for the development of precise predictive models (according to the Beer–Lambert law). In fact, the absorbance depends on the number of molecular bonds; thus, with a greater number, more molecular bonds can be excited [36]. Moreover, accurately predicting mineral content using infrared spectroscopy is challenging due to the absence of specific absorption bands in the infrared region [36]. Infrared spectroscopy primarily responds to bonds involving hydrogen, such as O-H, C-H, N-H, and S-H bonds [42]. Nevertheless, mineral content can still be predicted if it is bound to organic complexes or if it causes alterations in the water region of the spectrum [36]. These indirect associations can provide insights into mineral content, albeit with some limitations [36,42].

Wang et al. [43] investigated the determination of rare earth elements in soil samples using visible and NIRS from 400 to 1000 nm. Among the 15 rare earth elements studied by Wang et al. [43], which included Yb, only the models for neodymium and samarium demonstrated relatively good performance, with R^2^_CV_ values of 0.80 and 0.72, respectively, and RPD values of 2.37 and 1.94, respectively. However, the remaining models yielded weak results, with R^2^_CV_ values ranging from 0.6 to 0 and RPD values ranging from 1.7 to 1, with an RPD of 1.05 for the Yb developed model. The better results achieved in our study suggest that Yb may form complexes with organic components present in excreta but not in soils, thereby altering the absorption of these organic compounds [36].

While free metal ions like Yb and Ti exhibit limited infrared absorption due to their lack of dipole moment changes during vibrational transitions, their complexation with organic matter in excreta may alter their spectral detectability. According to Chen et al. [44], when metals are linked to organic ligands, particularly those containing carboxyl, hydroxyl, or aromatic groups, changes in the infrared spectra occur. These organic metal interactions modify the vibrational response detectable by NIRS. In our study, this mechanism may explain the relatively successful prediction of Yb in fecal samples compared to studies using soils [43], where such interactions are less prevalent or absent.

The existing literature on DMD prediction models in monogastrics primarily focuses on species other than poultry; however, there is a lack of research on poultry. For pigs, Bastianelli et al. [45] reported a less accurate DMD prediction model than our models for DMD_Yb_ and DMD_Ti_, with an R^2^_VAL_ of 0.60. However, Labussière et al. [33] achieved better results for growing piglets, with R^2^_VAL_ value above 0.85 and an RPD of 2.67. They also reported superior models for organic matter and nitrogen digestibility, with R^2^_VAL_ of 0.89 and 0.90, respectively, and RPD values of 3.05 and 3.11, respectively. In rabbits, Meiner et al. [46] achieved a similar performance (R^2^_CAL_ = 0.93; R^2^_CV_ = 0.79; SECV = 1.57) as the models presented in the current study for DMD_Yb_ and DMD_Ti_. On the other hand, Nuñez-Sánchez et al. [47] obtained a model with lower prediction ability for rabbits, as indicated by R^2^_CV_ and RPD values (0.65 and 1.69, respectively), compared to the ones reported in the present study. To the best of our knowledge, only Coulibaly et al. [25] have evaluated fNIRS for predicting DMD in poultry. Their results for DMD, starch, and protein digestibility showed lower performance, with an R^2^_CAL_ of 0.87 and an SECV of 2.26%, compared to the results of the present study for DMD_Yb_, DMD_Ti_, and DMD_ADF_.

Overall, studies conducted on ruminants have consistently demonstrated higher accuracy and improved performance compared to the findings reported in the literature and the results observed in our study involving monogastric animals. In goats, Glasser et al. [48] developed a prediction model with excellent precision for in vitro DMD, achieving an R^2^CV of 0.91 and an RPD of 2.5. Similarly, Coates and Dixon [49] successfully developed a robust prediction model for cattle, achieving an R^2^ of 0.83 and an associated RPD of 2.5.

## 5. Conclusions

This study confirmed the potential of fNIRS to estimate Ti and Yb concentrations in broiler excreta, supporting its use as a rapid screening tool for these elements. Among the four DMD markers evaluated (PEG, Yb, Ti, and ADF), only the Ti-based DMD model proved suitable for preliminary screening of digestibility. Regarding DMD, based on the ADL, it was not possible due to broiler diets generally containing less than 1% lignin. Moreover, NDF exhibited incomplete recovery, rendering the marker unsuitable for estimating digestibility. Results related to PEG calibration models indicated that, although feasible, they need to be tailored for each individual diet.

## Figures and Tables

**Table 1:** The composition of the experimental and adaptation diets administered to the broilers (%, as-fed basis).

Item	Adaptation Diet (d 1–15)	Experimental Diets (d 16–25)
Ingredients		
Corn	32.28	32.28
Wheat	40	-
Test ingredient ^1^	-	40
Extruded soybean	18	18
Processed animal protein ^2^	8	8
L-Lysine	0.34	0.34
DL-Methionine	0.31	0.31
L-Threonine	0.19	0.19
Isoleucine	0.13	0.13
Tryptophan	0.02	0.02
Salt	0.33	0.33
Vitamin and mineral premix ^3^	0.4	0.4
Titanium dioxide	-	2 g/kg
Ytterbium oxide	-	50 mg/kg
Polyethylene glycol	-	5 g/kg
In enzyme-supplemented diets		
Phytase (FTU)		1000 FTU/kg
Xylanase (BXU)		16,000 BXU/kg
β-glucanase (BU).		20,000 BU/kg
Calculated composition		
AME (kcal/kg)	3246	
Crude protein	19.6	
Calcium	0.48	
Phosphorus	0.5	

^1^ Each one of the 12 cereals was evaluated. ^2^ Derived from the processing of poultry products, 65% crude protein. ^3^ Provided per kg of feed: vitamin A (retinol acetate) 10,000 IU; vitamin D (vitamin D3) (cholecalciferol) 539–4800 UI; vitamin E/tocopherol) 45 mg; vitamin K3 (MNB, menadione nicotinamide bisulfate) 3 mg; vitamin B1 (tiamin mononitrate) 3 mg; 540 vitamin B2 (riboflavin) 9 mg; vitamin B6 (piridoxin chlorohydrate) 4.5 mg; vitamin B12 (cyanocobalamine) 0.04 mg; nicotinamide 51 mg; 541 pantothenic acid (calcium D-pantothenate) 16.5 mg; biotin (D−(+) biotin) 0.15 mg; folic acid 1.8 mg; choline chloride 350 mg; iron (iron 542 sulphate monohydrate) 54 mg; zinc (Zn, zinc oxide) 66 mg; manganese (Mn, manganese oxide) 90 mg; iodine (I, calcium iodine anhydrate) 543 1.2 mg; selenium (Se, sodium selenate) 0.18 mg; copper (Cu, copper sulphate pentahydrate) 12 mg.

**Table 2:** Descriptive statistics ^1^ of the calibration and validation databases.

Constituent ^2^	Calibration Set						Validation Set				
	*n*	Mean	SD	CV	Range		*n*	Mean	SD	CV	Range
Yb, g/kg	147	0.019	0.002	10.53	0.013–0.032		45	0.018	0.002	11.11	0.012–0.023
Ti, g/kg	147	0.503	0.057	11.33	0.320–0.730		45	0.485	0.054	11.13	0.310–0.800
DMD_Yb_, %	147	74.7	3.01	4.03	61.94–85.60		45	73.72	3.50	4.75	59.92–79.61
DMD_Ti_, %	147	74.20	2.89	3.89	59.96–82.27		45	73.35	3.37	4.59	58.25–78.23
DMD_ADF_, %	115	74.24	2.46	3.31	68.22–80.64		29	73.43	2.76	3.76	64.77–78.84

^1^ *n*, number of samples; SD, standard deviation; CV, coefficient of variation as a percentage. ^2^ DMD_Yb_, dry matter digestibility based on Yb content; DMD_Ti_, dry matter digestibility based on Ti; DMD_ADF_, dry matter digestibility based on acid detergent fiber content.

**Table 3:** Prediction models’ fitting statistics ^1^.

Constituent ^2^	Calibration Set							Validation Set					
	Mathematical Treatment ^3^	Scatter Correction ^4^	R^2^_CAL_	SEC	R^2^_cv_	SECV		R^2^_VAL_	SEP	Bias	Slope	RPD	RER
Yb	2.5.5.1	MSC	0.87	0.001	0.74	0.001		0.67	0.001	0	0.862	2.00	19.00
Ti	2.4.4.1	SNV + D	0.90	0.016	0.78	0.023		0.73	0.025	0	0.885	2.28	16.40
DMD_Yb_	2.4.4.1	SNV + D	0.89	0.960	0.75	1.43		0.68	1.56	0.019	0.863	1.93	15.17
DMD_Ti_	2.4.4.1	MSC	0.91	0.851	0.80	1.22		0.77	1.20	0.014	0.900	2.41	18.59
DMD_ADF_	2.4.4.1	SNV + D	0.90	0.793	0.66	1.41		0.25	2.06	0.440	0.477	1.19	6.62

^1^ R^2^_CAL_, coefficient of determination of calibration; SEC, standard error of calibration; R^2^_CV_, coefficient of determination of cross-validation; SECV, standard error of cross-validation; R^2^_VAL_, coefficient of determination in the external validation; SEP, error of prediction; RPD, ratio of performance to deviation; RER, range error ratio. ^2^ DMD_Yb_, dry matter digestibility based on Yb content; DMD_Ti_, dry matter digestibility based on Ti; DMD_ADF_, dry matter digestibility based on acid detergent fiber content. ^3^ Math treatment: derivative order, subtraction gap, first smoothing, second smoothing. ^4^ SNV, standard normal variate; D, detrend; MSC, multiple scatter correction.

## Data Availability

The raw data supporting the conclusions of this article will be made available by the authors upon request.
